# Multi-Robot Exploration of Unknown Space Using Combined Meta-Heuristic Salp Swarm Algorithm and Deterministic Coordinated Multi-Robot Exploration

**DOI:** 10.3390/s23042156

**Published:** 2023-02-14

**Authors:** Ali El Romeh, Seyedali Mirjalili

**Affiliations:** 1 Centre for Artificial Intelligence Research and Optimisation, Torrens University Australia, Brisbane 4006, Australia; 2 Yonsei Frontier Lab, Yonsei University, Seoul 03722, Republic of Korea; 3 University Research and Innovation Center, Obuda University, 1034 Budapest, Hungary

**Keywords:** robot path planning, optimization, algorithm, salp swarm algorithm, coordinated multi-robot exploration

## Abstract

Multi-robot exploration means constructing a finite map using a group of robots in an obstacle chaotic space. Uncertainties are reduced by distributing search tasks to robots and computing the best action in real time. Many previous methods are based on deterministic or meta-heuristic algorithms, but limited work has combined both techniques to consolidate both classes’ benefits and alleviate their drawbacks. This paper proposes a new hybrid method based on deterministic coordinated multi-robot exploration (CME) and the meta-heuristic salp swarm algorithm (SSA) to perform the search of a space. The precedence of adjacent cells around a robot is determined by deterministic CME using cost and utility. Then, the optimization process of the search space, improving the overall solution, is achieved utilizing the SSA. Three performance measures are considered to evaluate the performance of the proposed method: run time, percentage of the explored area, and the number of times when a method failed to continue a complete run. Experimental results compared four different methods, CME-GWO, CME-GWOSSA, CME-SCA, and CME, over seven maps with extra complexity varying from simple to complex. The results demonstrate how the proposed CME-SSA can outperform the four other methods. Moreover, the simulation results demonstrate that the proposed CME-SSA effectively distributes the robots over the search space to run successfully and obtain the highest exploration rate in less time.

## 1. Introduction

In mobile robotics, “exploration” refers to the operation when one or multiple robots work together to explore an unknown environment and quickly construct a finite map with various applications from indoor to outdoor settings. Multi-robot exploration of an unknown space is a fundamental task for mobile robot systems. A wide range of applications for mobile robot exploration using a group of robots can be mentioned, including autonomous transportation [1], industry [2,3], healthcare [4], rescue [5,6,7], and agriculture [8,9,10,11]. A robot must be able to navigate in a known or unknown environment [12] without human assistance. The most critical challenge for many mobile robots is to safely navigate from source to destination without colliding with their counterparts or other obstacles in the workspace. An unknown environment could be simple or complex and depends on the obstacle’s complexity. In both cases, exploring a simple or complex unknown environment starts with zero knowledge about the surrounding. Robots start the search operation without having any idea where obstacles are located and end up with a finite map. All the path planning applications could consider the exploration process as a first step, so that planning the next path becomes easier since the environment is known and has a map.

The main objective of this paper is to develop a new hybrid space exploration method that combines deterministic and meta-heuristic algorithms to quickly outline a definable map in an unknown environment. The proposed hybrid method combines deterministic coordinated multi-robot exploration (CME) and the SSA meta-heuristic. The rest of the paper is organized as follows:

Section 2 provides an in-depth literature review of the related work in path planning. Section 3 presents the proposed method. The results and discussions are given in Section 4. Finally, Section 5 concludes the work and suggests future directions.

## 2. Related Work

The single-robot exploration method was previously proposed based on the concept of frontiers between open space and unexplored space [13]. Another coordinated multi-robot exploration CME is based on a team of robots simultaneously exploring different regions of an unknown environment [14]. Both methods consider the cost of reaching a target point as well as its utility. The utility of the unfamiliar area visible from the target position is minimized whenever a target point is assigned to a specific robot. The most significant downsides of the centralized CME method are as follow, CEM is a deterministic approach and will always repeat the same search pattern; therefore, escaping local optima is not an option. The only way to solve this problem is to change the environment’s setting, which is impossible. Furthermore, ensuring a waypoint is not guaranteed, which has a huge impact on the robots as individuals, leading them to forget their assigned tasks and will result in a breakdown in coordination.

Deterministic CME is a popular approach that deterministically seeks to construct a definable map in an unknown space. A process of exploration aims to cover the entire environment in the least amount of time possible. The robots must, therefore, constantly monitor which portions of the environment have already been visited using a centralized technique [15]. To plan their trajectories and coordinate their operations [16], robots require the creation of a global map. The robots can always communicate with one another about their positions on the map and share the area they have investigated and the map itself so far. The occupancy grid [17] is used for communication between robots. Communication occurs by sensing and simultaneously searching the space and sharing their progress. Coordinated multi-robot exploration has been heavily used in mobile robot path planning [11,18,19,20].

In coordinated multi-robot exploration, a map is represented using the occupancy grid map [21]. The robot is given a standard vision of a 360-degree sensor view starting from its initial position. The robot is situated indoors and does not know its surroundings (unknown environment).

Robots use sensors to explore the unknown search space, gaining as much local knowledge as possible. It takes action to obtain the intended objective by learning from the sensor how to identify occupied spaces from unoccupied ones. The sensors’ detection range for the surroundings is limited. This is referred to as a frontier-based approach to exploring mobile robots, and this edge is known as a frontier [13]. The issue of allocating exploration assignments represented by frontier cells to the various robots is brought up because a map often comprises several unknown places. In case multiple robots are engaged in the search space, there must be a way to prevent them from moving to the same spot. In [22], multiple robots were used to explore and map an environment simultaneously. A global map was created by combining the robots’ individual maps. Frontiers were identified as the boundaries between explored and unexplored areas, and the robots navigated toward them using the frontier-based multi-robot approach. A robot operating system (ROS) was used for implementation, while the stage simulated the robots and their environments.

The above-mentioned papers show that the communication link between the robots breaks down as their distance grows, making it impossible for them to communicate quickly. In such circumstances, the centralized CME technique does not ensure clear waypoints on the map. This will have an impact on the tasks that have been allocated to certain robots and lead to a breakdown in coordination if any robots forget their tasks. Additionally, because CME is inefficient under all map conditions, an ideal solution can only be found by completely altering the map, which is not always feasible. Meta-heuristics can alleviate drawbacks as they are reliable optimization techniques.

Many researchers have addressed the problem of ground and aerial vehicle trajectory planning and obstacle avoidance using an optimization algorithm that mimics the behavior of living things such as fish, ants, bees, whales, wolves, and bats [23,24].

Over the past two decades, meta-heuristic optimization techniques have gained much traction. Most of them, such as genetic algorithm (GA) [25], ant colony optimization (ACO) [26], and particle swarm optimization (PSO) [27], are quite well-known among scientists from a variety of disciplines in addition to computer science. Such optimization techniques have been used in numerous academic fields of study in addition to the vast number of theoretical publications. Meta-heuristics have proliferated so widely because of four primary factors—simplicity, flexibility, derivation-free mechanisms, and local optima avoidance.

Meta-heuristics are rather easy to use. The sources of inspiration are frequently natural phenomena, animal behavior, or evolutionary theories such as the grey wolf optimizer (GWO) [28], antlion optimizer (ALO) [29], ant colony optimization (ACO) [24], sine cosine algorithm (SCA) [30], simulated annealing algorithm (SA) [31], salp swarm algorithm (SSA) [32], and gravitational search algorithm (GSA) [33]. Computer scientists can replicate various natural notions, suggest new meta-heuristics, combine two or more meta-heuristics, or enhance the existing meta-heuristics because of their simplicity. Additionally, the simplicity makes it easier for other researchers to readily learn meta-heuristics and use them to solve optimization problems.

The GWO algorithm is a bio-inspired algorithm that mimics grey wolves’ natural leadership structure and hunting strategy. Four different varieties of grey wolves, including alpha, beta, delta, and omega, are used to mimic the leadership hierarchy. The ALO algorithm imitates an antlion’s natural hunting strategy. There are five basic processes in the process of hunting prey: setting up traps, entrapping ants in them, catching prey, and setting up new traps. This optimizer was primarily inspired by moths’ natural transverse orientation navigational strategy. Moths use an extremely efficient system to fly at night that keeps them at a fixed angle to the moon and allows them to cover great distances in a straight line.

The salp swarm algorithm (SSA) is a novel bio-inspired algorithm proposed by Mirajalili et al. [32]. The SSA’s main inspiration is salps’ swarming behavior when navigating and searching the ocean, looking for food. The optimized SSA uses a mathematical model that mimics the social behavior of swarming salps; its functions generate many initial random candidate solutions and vary either outwards or towards the best answer. This algorithm additionally incorporates several random and adaptive variables to promote the exploration and exploitation of a search space at certain optimization milestones.

Typical stochastic algorithms have been found to have relatively low accuracy and randomness in the outcomes discovered, leading to near-optimal solutions. Although there are currently meta-heuristic approaches in space exploration, they have been heavily applied to single-robot applications [34] but limited in applications in multi-robot environments; thus, it is yet unclear how it will affect the multi-robot setup. Since meta-heuristic algorithms can produce distinctive solutions in real-time, it is therefore an attractive area for research in multi-robot environment settings.

A new collaborative coverage technique for mobile robot path planning based on a multi-robot system is provided in [35], by evaluating a cost function to maximize the exploration gain from the motion control. Additionally, a mechanism was designed to facilitate a collaborative map exploration for a single- and multi-robot system. A simulation successfully demonstrated the efficiency of the proposed technique, which produced a new deep space exploration method for rolling and jumping spherical robots [36]. The structure design was described, and its viable motion was tested through an established dynamic model. The system achieved a high level of performance and opened a future route of investigation for deep space exploration.

The main objective of this paper is to develop a new hybrid space exploration method that combines both deterministic and meta-heuristic algorithms to quickly outline a definable map in an unknown environment. The proposed hybrid method combines both deterministic coordinated multi-robot exploration (CME) and the meta-heuristic algorithm SSA. Related studies proposed by different researchers have been based CME [14], meta-heuristics [37], and hybrid methods combining meta-heuristic with deterministic algorithms [38,39,40]. Some studies focused solely on static sensor coverage faults and robot movements in uncharted surroundings [41,42,43] as well as exploration with deep reinforcement learning for mobile robots [44]; however, they coincide in meaning but the goal of both studies is to create a finite map. The rest of the paper is organized as follows:

Section 3 presents the proposed method. The results and discussions are given in Section 4. Finally, Section 5 concludes the work and suggests future directions.

## 3. Problem Formulation and Proposed Method

This paper proposes a new approach that combines the metaheuristic SSA with deterministic CME. Distant grids are used to divide up the entire map. The CME technique is used to assess the priority of neighboring cells. Each cell has its own utility and its cost value. CME takes care of assessing cells in relation to the robots. The total solution is then improved by optimizing the path using the SSA technique. Meta-heuristic SSA combined with deterministic CME can be considered a stochastic exploration that alleviates CME’s drawbacks. This study’s main formulated problem is exploring an unknown environment with a multi-mobile robot with sensor coverage to create a finite map. In the following paragraphs, CME is first proposed. There will be a discussion on the cost functions, SSA, and the process of hybridizing CME and SSA.

### 3.1. Coordinated Multi-Robot Exploration

The exploration procedure using more than one mobile robot, known as “multi-robot exploration,” starts with total ambiguity and ends with a defined map. Different algorithms are available for exploration based on the data obtained. Two approaches can be considered to create a map based on robot communication. The first is the centralized exploration approach, when all the robots share the same single map, and simultaneously sensing the environment enables them to keep track of each other’s progress. Second is the decentralized approach, based on individual map construction [45]. Data exchange needs to be coordinated only when robot positions overlap. In this paper, the centralized technique is used, which calculates the utility values that all robots update through iterations, as well as the real-time cost of travelling for each robot.

**Figure 1:**
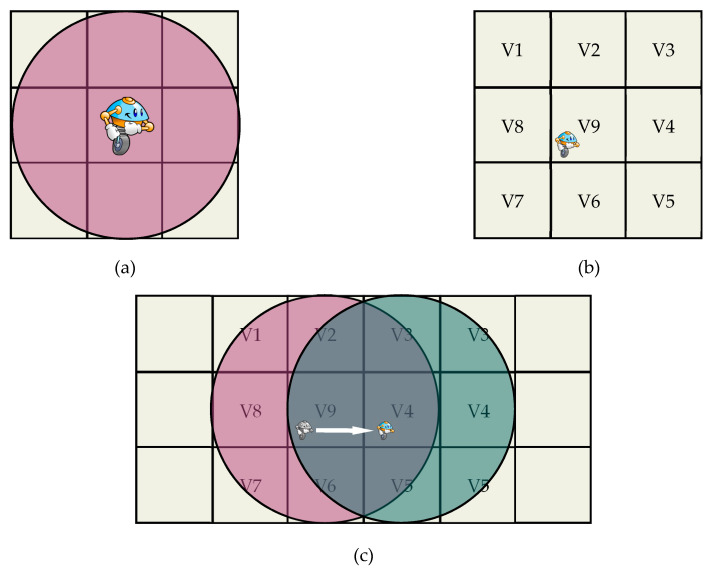
Senor view range in the grid cells: (**a**) sensor range cover $\left[V1,\ldots ,\;V8\right]$ around the robot; (**b**) eight cells around the robot and cell 9 is the robot position; (**c**) robot moving from right to left showing the sensor range does not cover the cost V1, V7, V8.

In coordinated multi-robot exploration, a map is represented using an occupancy grid map [21]. The robot is given a standard vision of a 360-degree sensor view starting from its initial position. The robot is situated in an indoor setting, and it does not know its surroundings (unknown environment). The occupancy grid map stores the utility and the cost of traveling in its cells as numerical values, showing the likelihood that an obstruction occupies the grid cell. Because of the limitation of sensor range to cover the entire space and find an optimal path at once for the robot, it is used to sense the frontier cells [46,47]. Frontier cells are the most important objective when it comes to building a finite map in an unknown space [48]. On the occupancy grid map, only nine cells are covered by a robot with a sensor view (Figure 1).

#### 3.1.1. Cost Function

The best route from the robot’s present position to all frontier cells is computed using a deterministic version of the value iteration to calculate the cost of reaching the current frontier cells. Through initialization (Equation (1)), occupancy grid probability, sensor view, and Euclidean distance (Equation (2)), when a cell has already been discovered, the prior step’s cost for this cell is added to the current position’s cost. Otherwise, the cell is designated as a frontier cell without the backward costs of the earlier phases of the ray beams which open it predominantly (Equation (3)). The *x-th* cell in the direction of the *x*-axis and the *y-th* cell in the direction of the *y*-axis of the two-dimensional occupancy grid map are represented by the tuple $\left(x,\;y\right).$ The cost of moving through a grid cell $\left(x,\;y\right)$ is inversely correlated with its occupancy probability value $P(occ_{xy})$. The following two steps are used to calculate the minimum cost path [14]:

1. Initialization.

(1) $$V\left(x,y\right)=\left\{\begin{array}{c} 0,\;\mathrm{if}\;\left(x,\;y\right)\;\mathrm{is}\;\mathrm{the}\;\mathrm{robot}\;\mathrm{position}\\ \infty \;,\;\mathrm{Otherwise} \end{array}\right.$$

2. Update loop for all grid cells (*x*, *y*).

(2) $$V\left(x,y\right)=min\left\{V_{x+\Delta x,y+\Delta y}+(\sqrt{\Delta x^{2}+\Delta y^{2}}\;)\times \;P(occ_{x+\Delta x,y+\Delta y})\right\}$$

(3) $$V\left(x,y\right)=min\left\{\left(\sqrt{\Delta x^{2}+\Delta y^{2}}\;)\times \;P(occ_{x+\Delta x,y+\Delta y}\right)\right\}$$

where $P(occ_{xy})$ is the maximum occupancy probability value of a grid cell the robot can travel through, Δx, Δy ∈ [−1, 0, 1], and $P(occ_{x+\Delta x,y+\Delta y})$ ∈ [0, *max*(*occ*)] and *max*(*occ*) is the maximum occupancy probability. Three possible values are assigned to the occupancy probability [0, 0.5, 1]. A zero value explains that the cell is assumed empty and free of obstacles. A value of 0.5 is the probability of an unknown cell compared to the cell occupied by a barrier, which is about equal to one. Finding the lowest value across neighboring cells is ideal for a robot moving to the next position. A single mobile robot system may just need to conduct a low-cost search to locate itself. However, the interaction of the collective organization is necessary for the multi-robot system throughout the investigation. The CME technique introduced a capability for workload distribution among robots [14].

#### 3.1.2. Utility Value

Utility value is a number assigned equally to each cell in a grid map that measures if a cell has been utilized. Every grid cell on the map has the same initial utility values. As can be seen in Equation (4), the utility values of the robots’ frontier cells decrease as they explore the map toward a new position. Robots ignore visiting cells with few utilities and only explore new positions on a map by going to grid cells with greater utility values. In order to optimize utility values, the robots look for new places they have not yet visited. The cell cost for each robot is proportional to the distance between the robot and the cell. The utility of a frontier cell depends on how many robots are migrating to it or an area nearby.

(4) $$U_{i}^{cell}=U_{i-1}^{cell}-\overset{n-1}{\underset{i=1}{\sum }}P\left(\left\|occ_{x,y}^{c}-occ_{x,y}^{r}\right\|\right)$$

where $U_{i}^{cell}$ is the utility of the current grid cell, $U_{i-1}^{cell}$ is the utility of the same cell in the previous stat during the exploration process, and P represents the probability of the current cell *i*. The utility of the maximum value is iteration *i*, as in Equation (5).

(5) $$\left(i,cell\right)=argmax\;\left\{U_{i}^{cell}-V_{x,y}\right\}$$

The maps’ dimension is maintained at 20 m × 20 m, and the sensor ray length is 1.5 m. On the maps, the white region denotes the area that has been examined, while the dark-occupied region indicates the presence of an obstruction. To aid cooperative exploration, to diverge the directions of the robots at the start, their initial positions must be close to each other, and this way their sensors scan each other, and the next move will result in decreasing utilities of the selected targets and a divergence in different directions (Figure 1).

### 3.2. Salp Swarm Algorithm

**Figure 2:**
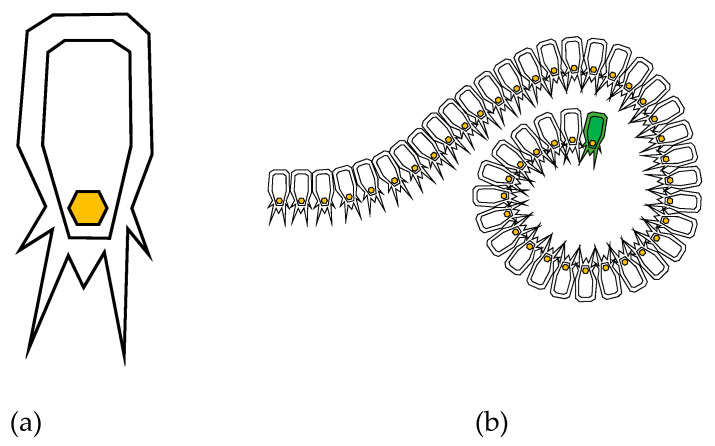
(**a**) Individual salp and (**b**) swarm of salps (chain).

The salp swarm algorithm (SSA) is a meta-heuristic algorithm recently proposed by Mirjalili et al. [32]. SSA is a swarm intelligence technique that mimics the intelligence of salp swarms in nature. This algorithm (Algorithm 1) was inspired by the collective behavior of a group of salps as a chain. Salps belong to the family of Salpidae and have a transparent barrel-shaped body. Their tissues and movements are very similar to jellyfish. Figure 2a depicts the structure of a salp. In deep oceans, salps often form clusters called a salp chain (Figure 2b). This swarming behavior helps salps to quickly coordinate changes and find more food. SSA has the advantages of fewer parameters and easy implementation.

The primary characteristic of SSA that differentiates it from other well-known swarm meta-heuristic algorithms is its hierarchical chain, as shown in Figure 2b. The salp population is initially divided into two family groups: the leaders and followers. The salp at the front of the chain is the leader, while the other salps are referred to as followers. These salps have a leader who directs the swarm, and the followers follow each other and obey the leader. The location of the salp is characterized by an n-dimensional search area where n is the no. of variables. The food source F is the target for the salp in the search boundary.

To update the position of the leader, the following equation is represented by:

(6) $$x_{j}^{1}=\left\{\begin{array}{c} F_{j}+c_{1}\left(\left(ub_{j\;}-lb_{j}\right)c_{2}+lb_{j}\right)\;\;\;c_{3}\geq \;0.5\\ F_{j}-c_{1}\left(\left(ub_{j\;}-lb_{j}\right)c_{2}+lb_{j}\right)\;\;\;c_{3}<0.5 \end{array}\right.$$

where $x_{j}^{1}$ *j* and $F_{j}$ denote the positions of the leader and food source, respectively, in the jth dimension. The leader updates its position with respect to the food source. $ub_{j\;}\mathrm{and}\;lb_{j}$ are, respectively, the upper bound and the lower bound of the jth dimension. $c_{1},\;c_{2},\;\mathrm{and}\;c_{3}$ are random numbers.

(7) $$c_{1}=2e^{-{\left(\frac{4i}{MaxIter}\right)}^{2}}\;c_{2},\;c_{3}\;\in \;[0,\;1]$$

where i is the current iteration and MaxIter is the maximum number of iterations. $c_{2}$ and $c_{3}$ are numbers in the interval of [0, 1] and are randomly generated. The presence of random vectors $c_{1}$,$c_{2}$, and $c_{3}$ makes the SSA meta-heuristic. They provide the step size and whether the next position in the jth dimension should be towards positive or negative infinity.

Newton’s law of motion is used in the following equations to update the positions of the followers:

(8) $$x_{j}^{i}=\frac{1}{2}at^{2}+v_{0}t$$

where i is the current iteration when i ≥ 2, $x_{j}^{i}$ is the position of the ith follower salp in the jth dimension, $v_{0}$ is the initial speed and t is the time. The parameters a and $v_{0}$ are found, respectively, by:

$$a=\frac{v_{final}}{v_{0}},\;v_{0}=\frac{x-x_{0}}{t}$$

Time in optimization is considered to be iteration and $v_{0}=0$ equal to 1 because the discrepancy between iterations equals 1; therefore, Equation (8) can be modified to:

(9) $$x_{j}^{i}=\frac{x_{j}^{i}+x_{j}^{i-1}}{2}\;\;i\geq \;2$$

where i is the current iteration when i ≥ 2, $x_{j}^{i}$ is the position of the ith follower salp in the jth dimension.
**Algorithm 1** Salp Swarm Algorithm SSA1: $Initialize\;the\;salp\;population\;Xi\;\left(i=1,\;\ldots ,\;n\right)\;considering\;upper\;and\;lower\;bounds$2:     while iteration is not over do
3: $\;\;\;\;\;\;\;Calculate\;the\;cost\;function\;of\;each\;search\;agent\;\left(salp\right)$
4:        Food=the best search agent
5: $\;\;\;\;\;\;\;Update\;c1\;by\;Equation\;\left(7\right)$
6: $\;\;\;\;\;\;\;for\;each\;salp\;\left(Xi\right)$7:        if (i<=Xi/2)
8:            *Evaluate Equation (6) to update the salp leader position*9:            *else*10:          *Evaluate Equation (9) to update the position of the salp follower*11:            *end*12:         *end*13:        *Amend the salps based on the upper and lower bounds of variables*14:    *end while*

The SSA’s ability to direct salps towards the food source while updating it through iterations illustrates such a strong optimization that it may be applied among a wide range of fields. SSA’s hierarchical structure is an additional element that sets it apart from other well-known swarm intelligence algorithms in terms of effectiveness. In this paper, the SSA is employed to solve the exploration problem for the multi-mobile robot system. The main benefit of the SSA is the high probability of local optima avoidance in the context of composite functions. This is because the SSA stores the best solution produced thus far and assigns it to the food source variable. Thus, the best solution never gets lost, even if the whole population deteriorates. Additionally, it has only one main controlling parameter ($c_{1}$), decreasing adaptively throughout iterations, first exploring the search space and then exploiting it. This is in addition to the primary advantages of SSA; characteristics include the simplicity of its implementation due to its structure as well as its low computational requirement compared to other techniques. Additionally, it has a faster convergence rate due to a continuous search space reduction and lower judgement parameters (leaders and followers). Potentially, all the above-mentioned benefits of SSA play a great role in outperforming other algorithms, such as GWO, WOA, MFO, ABC, SCA, etc.

### 3.3. Hybrid CME-SSA

The process has been optimized to build a finite map with a multi-robot system using the meta-heuristic SSA. It generates random parameters that help the robot with its next move by identifying the robot’s best next positions. Consequently, the SSA selects the robot’s next move position. When the CME cannot continue real-time processing, SSA, without prior information about the environment, can continue and find the best optimal solutions for exploring an unknown space for mobile robot sensor systems.

Algorithm 2 is the pseudo-code of the proposed hybrid method. Initially, the grid map is initialized with a value equal to 1 as a utility for the whole cells. Only eight cells around the robot that are covered by the sensor, as shown in Figure 1a, are the candidates for the next move, and only one cell can be selected. Since each cell has a cost and utility value, CME computes the cost and deducts the utilities from the cost of the surrounding 8 grid cells using Equation (5). Then, the proposed stochastic method defines 4 maximum utility values as four leaders and assigns them. The priorities between the 4 leaders changes based on the random ($c_{1}$ and $c_{2}$) Equation (7), and the values of occupancy probability of the four dominated cells.

(10) $$X_{salpleader,i}=\left\{\begin{array}{c} P_{i}\left(occ_{x+\Delta x,y+\Delta y}\right)+c_{1}\left(\left(ub_{i\;}-lb_{i}\right)c_{2}+lb_{i}\right)\;\;\;c_{3}\geq \;0.5\\ P_{i}\left(occ_{x+\Delta x,y+\Delta y}\right)-c_{1}\left(\left(ub_{i\;}-lb_{i}\right)c_{2}+lb_{i}\right)\;\;\;c_{3}<0.5 \end{array}\right.$$

where $X_{salpleader,i}$ i denotes the positions of the leader and food source, respectively, in the ith dimension. $P(occ_{x+\Delta x,y+\Delta y})$ is the maximum occupancy probability value of a grid cell the robot can travel through, Δx, Δy ∈ [−1, 0, 1], and $P(occ_{x+\Delta x,y+\Delta y})$ ∈ [0, *max*(*occ*)] and *max*(*occ*) is the maximum occupancy probability. The leader updates their position with respect to the $P(occ_{x+\Delta x,y+\Delta y})$. $ub_{i}$ and $lb_{i}$, which are, respectively, the upper bound and the lower bound of the ith dimension. $c_{1},\;c_{2}\;\mathrm{and}\;c_{3}$ are random numbers.

The grids orientation function is as follows:

(11) $$gridsOrientation\;\left(x,y\right)=\left[V_{1},\;V_{2},\;V_{3},\;V_{4},V_{5},\;V_{6},\;V_{7},\;V_{8},\;V_{9}\right]$$

where the decision variable x, y is the current robot position and $V_{1}$,…, $V_{9}$ are the costs of the nine cells around the robot, including the cost of its current position using Equations (1)–(3).
**Algorithm 2** Coordinated Multi-Robot Exploration with Salp Swarm Algorithm CME-SSA*1:  Initialization*$\left\{\begin{array}{c} Number\;of\;Robots\;N,\;sensor\;range\\ Iteration\;i,\;Initial\;position\\ Set\;the\;utility\;of\;all\;cells\;to\;1 \end{array}\right.$*2:* While iteration is not over do *3:*    For N robot*4:*$\;\;\;\;\;\;Set\;coordinates\;of\;cost\;V_{Cell}\;$*5:*$\;\;\;\;\;\;\;\;\;\;Calculate\;cost\;of\;\;V_{Cell}$*6:*       *Update* $Utility_{cell}^{iteration}$*and cost of*$V_{Cell}$*7:*       *Calculate* $c_{1}$*,*$c_{2}$*and*$c_{3}$*8:*       *Find leaders* $salpLeader_{1}$*,*$salpLeader_{2}$*,*$salpLeader_{3}$*,*$salpLeader_{4}$*(Line 7 Algorithm 1)**9:*       *Find* $X_{Leader1}$*,*$X_{Leader2}$*,*$X_{Leader3}$*,*$X_{Leader4}$*10:*       *Find the next position for* $Robot_{i}$*as max (*$X_{Leader1}$*,*$X_{Leader2}$*,*$X_{Leader3}$*,*$X_{Leader4}$*)**11:*       *Reduce utility on a new position**12:       end for**13:  end while*

Driving towards the source of food is defined by Equation (10). In the SSA, Newton’s law of motion is used to update the positions of the followers, which is related to the natural behavior of the salp swarm. However, in the proposed method, it is not required as the selecting process is based on choosing the maximum $X_{salpleader,1}$, $X_{salpleader,2}$, $X_{salpleader,3}$ or $X_{salpleader,4}$.

After the robot makes the next move based on the maximum value of the leaders’ positions (line 10 in Algorithm 1), Equation (4) is used to reduce the utility value of the neighbor grid cells. Then, the randoms ($c_{1}$, $c_{2\;}\mathrm{and}\;c_{3}$) are regenerated using Equation (7) for the subsequent iteration. The proposed CME-SSA method aims to explore the unexplored areas in the grid map. That is because the utility of the unexplored cells has greater values which makes them more attractive to the salps’ leader to select more than the explored ones, and since there are four candidates to select from, the chances of exploring new different areas with each iteration are high. Additionally, it should be noted that the computational complexity is $O\left(Iter\left(d\times n+CostF\times n\right)\right)$ where Iter is the number of iterations, n is the number of solutions, d is the dimension and CostF is the cost of the objective function.

The main benefit of the CME-SSA is the high probability of local optima avoidance because one of the SSA’s advantages is represented by the implemented memory feature that allows SSA to save the best optimal solution obtained to date and set it as the best food source. Thus, the best solution of the CME-SSA never gets lost. Additionally, exploration and exploitation over the number of iterations are well controlled because of one main parameter (*c*_1_). Moreover, it is easy to implement and has lower memory requirement compared to other techniques, making the exploration process achievable in less time. Finally, because the SSA mimics the salp’s behavior in the chain of leaders and followers, it is easier for the robots to explore tight corridors and corners efficiently. Its main cons are when the exploration space is free of obstacles, it requires extra 10 to 20 iterations to explore most of it. 

## 4. Results and Discussions

Several test cases should be employed to confirm an algorithm’s performance in the optimization field using bioinspired and meta-heuristics algorithms. This is due to the stochastic nature of these algorithms, in which a proper and sufficient set of test maps is divided into two categories. The simple environment consists of at least one obstacle along with the map borders, and the complex environment consists of more obstacles. This is to test the exploration in a different environment.

This section presents the simulation of the proposed CME-SSA method. Two different environments, with different complexities, are being utilized to assess the algorithm’s performance. The map complexity can be changed by including barriers and altering their relative direction. To prove the efficiency of the proposed method CME-SSA, four other algorithms are used:

- Original coordinated multi-robot exploration (CME);
- Coordinated multi-robot exploration and grey wolf optimizer algorithms (CME-GWO);
- Coordinated multi-robot exploration and the sine cosine algorithm (CME-SCA);
- Coordinated multi-robot exploration and grey wolf optimizer algorithms combined with salp swarm algorithm (CME-GWOSSA) has been implemented under similar conditions.

The results of the proposed method CME-SSA are compared then with CME-GWO, CME-GWOSSA, CME-SCA, and the original deterministic CME, to analyze and determine its potential benefits.

**Figure 3:**
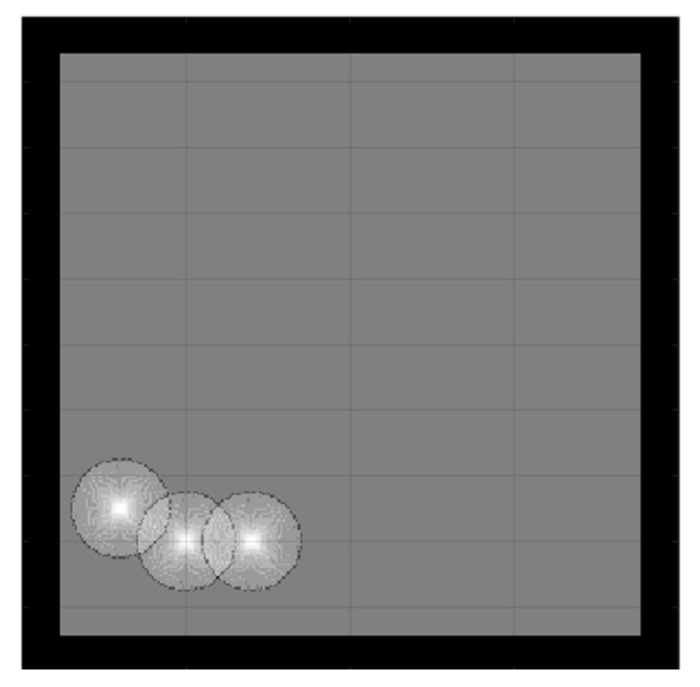
Initial position of coordinated exploration of three robots.

To ensure a fair comparison between all algorithms, in the simulation section, the dimension of all the maps was maintained the same at 20 m × 20 m. On the maps, the white region denotes the area that has been examined, while the black zone indicates the presence of an impediment. To aid cooperative exploration, and to diverge the directions of the robots at the start, their initial positions were close to each other so that their sensors scanned each other, and the next move resulted in decreasing utilities of the selected targets and a divergence in different directions (see Figure 3).

Because the robots can move in any direction arbitrarily, the main goal of this simulation analysis was to take care of and obtain the percentage of the explored area using a different approach. The percentage of the explored area was calculated using the following Equation (11) ($Total_{egc\;})$.

(12) $$Total_{egc\;}=\frac{T_{unexp_{u}}-T_{exp_{u}}}{T_{unexp_{u}}}\;\times \;100$$

where $Total_{egc}$ is the total explored grid cells, and possible values vary between 0% and 100%. Zero is the minimum value where the area is not being explored, and 100% is the maximum value and represents a fully explored area. $T_{unexp_{u}}$ represents the value of unexplored utility values in the obstacle-free zone, while $T_{exp_{u}}$ represents the explored utility values. Three different key aspects were considered for the comparison between the proposed CME-SSA method and the other methods after the simulation was completed: $Total_{egc\;}$, time consumption for exploring a map, and the number of cases where a technique failed to complete a full run.

The same parameters were set to use across the different approaches used in this simulation. The number of iterations, obstacles positions, number of robots and their initial positions, map dimension, and sensor range was the same for all algorithms and across all simulations to ensure a fair comparison.

**Figure 4:**
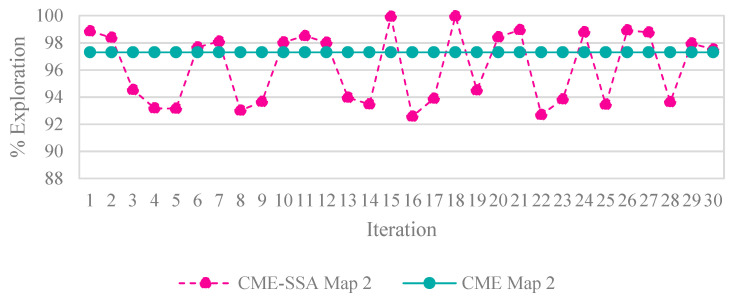
Percentage of exploration per iteration for CME-SSA and CME.

**Figure 5:**
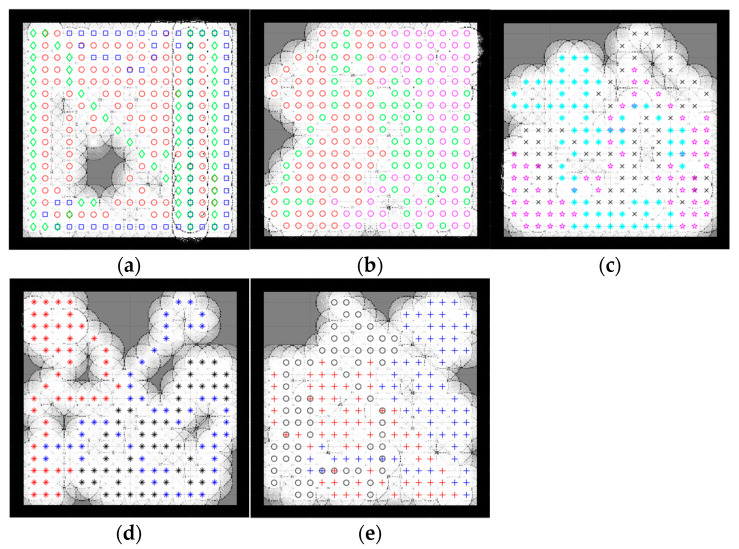
Percentage of the explored area after implementing the CME-SSA and the other approaches on simple map 1. Simple map 1: (**a**) CME 93%, (**b**) CME-SSA 90%, (**c**) CME-GWO 84%, (**d**) CME-SCA 73%, (**e**) CME-GWOSSA 80%.

**Figure 6:**
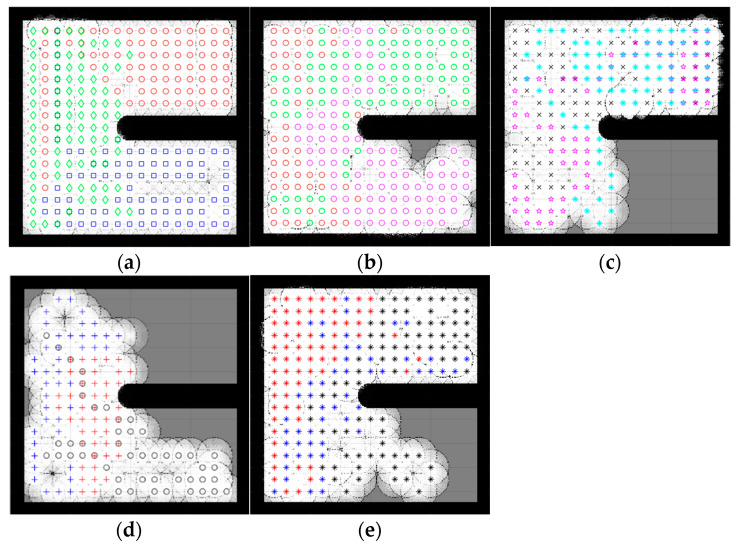
Percentage of the explored area after implementing the CME-SSA and the other approaches on simple map 2. Simple map 2: (**a**) CME 97%, (**b**) CME-SSA 92%, (**c**) CME-GWO 72%, (**d**) CME-GWOSSA 61%, (**e**) CME-SCA 74%.

Because the optimization algorithms are stochastic and cannot run forever to obtain reliable results, the population was also picked randomly; therefore, to ensure that the results were optimized correctly across multiple algorithms, a strategy based on the central limit theorem [49], which suggests that single-problem and multi-problem analyses are frequently used to contrast the findings of computational intelligence experiments, highlighted sample sizes of around 30 to 50 randomly, which were deemed sufficient for the distribution to be fairly normally distributed. A sample size of 30 is the minimum number to be considered a normal distribution; hence, in this simulation, the sample size was 30. Samples were collected by running each algorithm with 100 iterations 30 times. Taking into account the new stochastic technique must find the optimal result. Therefore, the number of samples was 30. Since SSA is stochastic, it should be noted that each run generated different results. In contrast, the deterministic CME simply needs to run once, meaning that the robot’s motion trajectory remains unchanged when the examined areas are unchanged, Figure 4 illustrates the stability and constancy of the CME simulation over the course of the 30 runs. Each color on the map represents a single robot regardless of the symbol (Figure 5 and Figure 6) same applies to the rest of the maps in complex environments. Since three robots were used in this simulation, there will be three different colors to differentiate between them.

### 4.1. Simple Map

A simple map is an environment almost free of obstacles. In this simulation, two types of simple maps were introduced (Figure 5 and Figure 6). This type of environment makes it more feasible for robots to make easy moves and stay away from each other because the map has fewer obstacles and more free spaces.

Figure 5 and Figure 6 illustrate the exploration results obtained from five different algorithms, including one deterministic, where each path color indicates a different robot. Despite the slightly better exploration provided by CME in the exploration average (which shows 93% in simple map 1 and 97% in simple map 2) than the proposed method, including the other different approaches, Figure 4 shows how CME-SSA outperformed CME 16 times with better results; the rest were very close. Different results were obtained through the stochastic approaches.

Due to the random values of C1, C2, and C3 in the SSA, the total exploration values differed in each run. After 30 completed simulations, with 100 iterations each, the proposed CME-SSA approach achieved an exploration average of 89.72% in simple map 1 with an std of 3.1 and 96.21% in simple map 2 with an std of 2.9. However, the exploration results of the other stochastic methods are as follows and in Table 1:

- CME-GWO showed an exploration average of 88.44% in simple map 1 with an std of 2.5 and 91.7% in simple map 2 with an std of 3.2
- CME-GWOSSA provided an exploration average of 87.84% in simple map 1 with an std of 5.2 and 87.63% in simple map 2 with an std of 7.4
- CME-SCA delivered an exploration average of 82.94% in simple map 1 with an std of 7.4 and 88.48% in simple map 2 with an std of 8.3

In the deterministic CME method, the robots always make the next move decision based on the maximum utility value, so it follows the same pattern every time. Therefore, the same results will be generated when running CME more than one time. In the hybrid stochastic method, the output results vary with each run due to the random parameters used in their equations. The prediction of the next move for a robot depends on the utility value stored in each cell, which sometimes results in obstacle collision. This issue occurs when two or more cells that are candidates for the next move have the same utility value, and at least one of them is occupied by an obstacle; however, because SSA continuously generates random C1, C2, and C3 parameters, it helps CME-SSA to find and select the best, new, less risky position.

In Figure 5, simple map 1 shows the implementation of CME-SSA in an obstacle-free area, and then the obstacle is introduced in Figure 6, which is simple map 2.

As shown, the proposed CME-SSA has greater map coverage than the original CME, at least 16 times larger. Additionally, Table 1 shows how the percentage average of the proposed CME-SSA method’s explored area was greater than all the other stochastic approaches: CME-GWO, CMEGWOSSA, and CME-SCA. It has also been observed that the proposed approach performs even better when the number of iterations is raised to 110 or 130.

Table 2 shows the average time spent in seconds for each approach to complete one full run (100 iterations). Additionally, it is clear that the proposed method showed an average of 10.9 s for map 1 and 11.01 s for map 2, which was the minimum time compared to the other methods, including CME.

The number of failed simulations across two simple maps in Figure 5 and Figure 6 are presented in Table 3. A single run that could not complete 100 iterations successfully due to neighbor cells being occupied by obstacles or another robot is considered a failed simulation. The number of failed runs for the proposed stochastic method CME-SSA was zero for both simple map 1 and map 2. Additionally, this shows how the combined deterministic meta-heuristic CME-SSA gave its best performance in simple complexity environments (map 1 and map 2).

### 4.2. Complex Space Map

**Figure 7:**
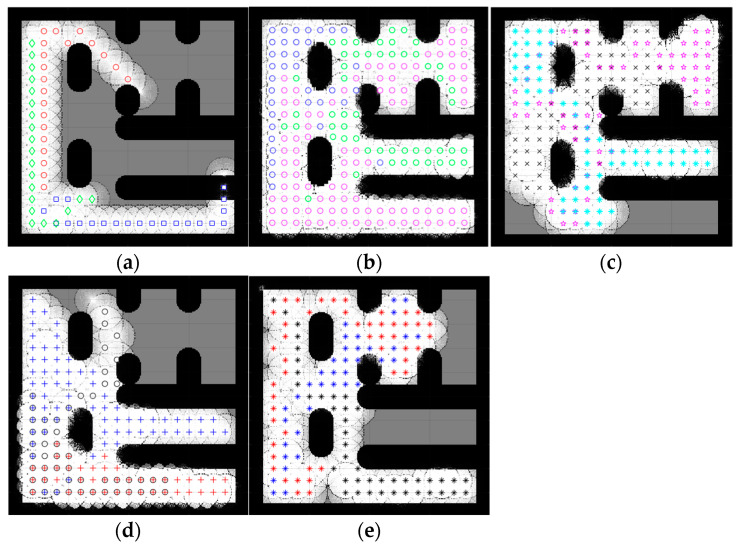
Percentage of the explored area after implementing the CME-SSA and the other approaches on complex map 1. Complex map 1: (**a**) CME, 39.24% (Iteration 21); (**b**) SSA, 98.36%; (**c**) CME-GWO, 86.88%; (**d**) CME-GWOSSA, 71.47%; (**e**) CME-SCA, 74.56%.

**Figure 8:**
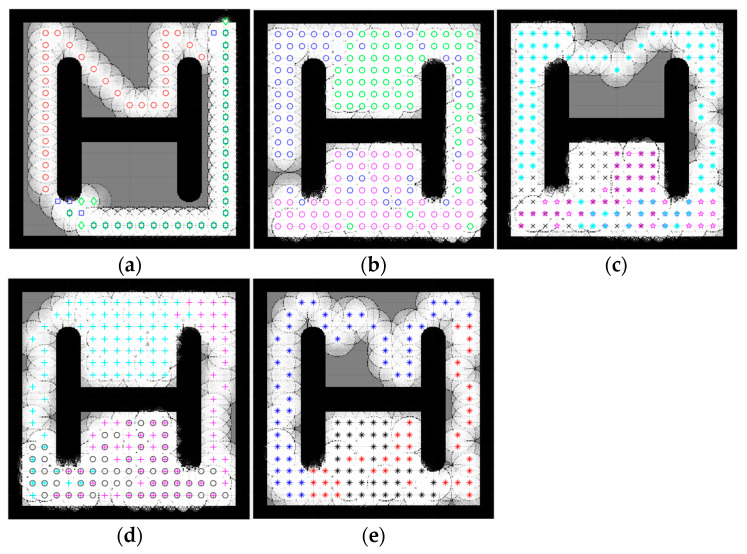
Percentage of the explored area after implementing the CME-SSA and the other approaches on complex map 2. Complex map 2: (**a**) CME 52.16% (Iteration 33); (**b**) CME-SSA, 96.45%; (**c**) CME-GWO, 80.61%; (**d**) CME-GWOSSA, 89.13%; (**e**) CME-SCA, 76.57%.

**Figure 9:**
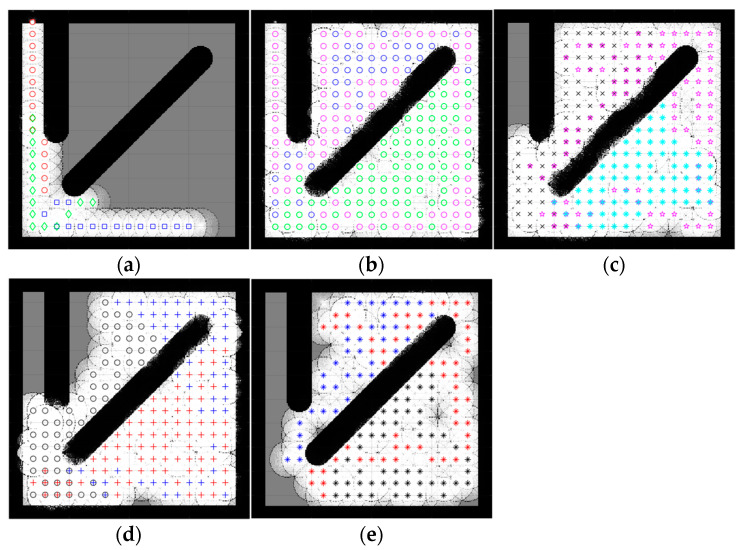
Percentage of the explored area after implementing the CME-SSA and the other approaches on complex map 3. Complex map 3: (**a**) CME, 36.48% (Iteration 15); (**b**) CME-SSA, 98.49%; (**c**) CME-GWO, 90.51%; (**d**) CME-GWOSSA, 86.32%; (**e**) CME-SCA, 78.44%.

**Figure 10:**
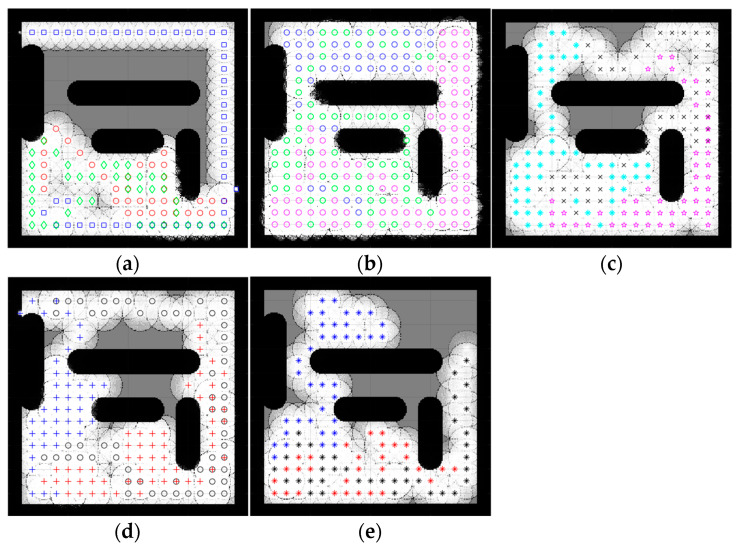
Percentage of the explored area after implementing the CME-SSA and the other approaches on complex map 4. Complex map 4: (**a**) CME, 69.45% (Iteration 50); (**b**) CME-SSA, 97.25%; (**c**) CME-GWO, 86.57%; (**d**) CME-GWOSSA, 81.44%; (**e**) CME-SCA, 79.12%.

**Figure 11:**
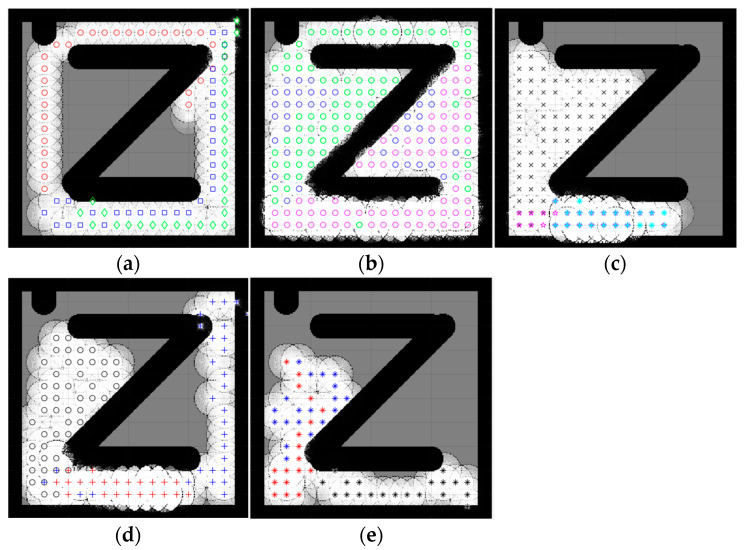
Percentage of the explored area after implementing the CME-SSA and the other approaches on complex map 5. Complex map 5: (**a**) CME, 54.23% (Iteration 31); (**b**) CME-SSA, 96.59%; (**c**) CME-GWO, 53.85% (Iteration 51); (**d**) CME-GWOSSA, 58.32% (Iteration 58); (**e**) CME-SCA, 44.56% (Iteration 49).

An environment with more than one obstacle is considered a complex map. In this section, the five complex maps were introduced to evaluate the performance of the proposed CME-SSA method against the deterministic CME and the other hybrid meta-heuristic methods: CME-GWO, CME-GWOSSA, CME-SCA (as shown in Figure 7, Figure 8, Figure 9, Figure 10 and Figure 11).

Low map coverage was recorded for the deterministic CME over the five complex maps (Figure 7, Figure 8, Figure 9, Figure 10 and Figure 11). This algorithm could not complete the full 100 iterations on any of them, possibly due to neighbor cells being occupied by obstacles and another robot. Because of the nature of the deterministic algorithm, when given a specific input, it will always produce the same output passing through the same set of states; therefore, improvement in the next iteration cannot happen. In contrast, it is evident from Figure 7, Figure 8, Figure 9, Figure 10 and Figure 11 that the proposed hybrid stochastic CME-SSA approach produces better results than the other meta-heuristic hydride techniques: CME-GWO, CME-GWOSSA, and CME-SCA.

Different types of narrow tunnels and corridors were introduced in the five complex maps. The proposed CME-SSA moved efficiently between them with the highest coverage rate. Although complex map 1 (Figure 7) has more obstacles than the others and small zones, CME-SSA was able to explore it with a rate of 98.36%, as well as map 2 (Figure 8) with a rate of 96.45%. Moreover, another crucial factor in map 3 (Figure 9) was the tight tunnel on the upper-left side of the map, which was only explored successfully by the proposed CME-SSA, and the total coverage was 98.49%. Similarly, in map 4 (Figure 10), with narrow corridors, the result was 97.25%, and the CME-SSA could efficiently explore the middle zone. Lastly, map 5 (Figure 11) was one of the most difficult environments because the initial positions of the robots were very close to the barriers. Additionally, the tiny tunnel made it very hard for them to escape and not collide with each other, which caused a high number of failures to continue the 100 iterations successfully in the the CME-GWO, CME-GWOSSA, and CME-SCA, but not the CME-SSA with 96.59% of coverage. Table 4 summarizes the results and shows the average performance of each method and how stable it is through the average and standard deviation. The proposed hybrid stochastic CME-SSA showed an innate ability to maneuver around obstacles, tunnels, and corridors without getting stuck.

### 4.3. Results, Analysis, and Discussion

The proposed CME-SSA delivered a high space exploration, and the qualitative results demonstrated that; however, they could not measure its efficiency. Therefore, in this section, two performance indicators have been employed to measure the efficient performance of CME-SSA. Average and standard deviation are the two indicators. Furthermore, the same indicators have been applied on four different algorithms after successfully completing a full run of 100 iterations, independently repeated 30 times. The average indicator shows the explored area’s average, which explains the mediocre performance. In contrast, the standard deviation indicator shows the proposed method’s stability compared to similar algorithms (Table 4).

Average and standard deviation are not able to measure individual runs. They can only measure the overall performance. Comparing single runs and ensuring the significance of the results is very important, which can be achieved through a statistical test. Therefore, this section uses the Wilcoxon rank-sum test to efficiently compare and analyze the statistical results. Two hypotheses are defined H0 and H1. H0 assumes the exploration rate and the time consumption obtained by the proposed CME-SSA fall behind the other four methods, whereas H1 assumes that the CME-SSA outperformed the other methods. A *p*-value of less or equal to 0.05 is statistically significant to reject the null hypothesis. The statistical test was applied as follows: The best method’s results in each test function were chosen and independently compared with other methods. For instance, if CME-SSA results were the best, then a pairwise comparison was performed between CME-SSA and CME-GWO, CME-SSA and CME-SCA, and so on. Throughout the paper, the same approach was used.

The same test maps utilized in the previous section were employed here but in a different complexity setup. Considering that the CME deterministic algorithm could not complete a full run on each map in the complex environment, that was considered strong evidence against the null hypothesis. Therefore, the *p*-value was not calculated in Table 5.

The hybrid CME-SSA was compared against a different set of hybrid methods of CME-GWO, CME-GWOSSA, CME-SCA, and the original deterministic algorithm CME. To ensure a fair comparison between all algorithms, in the simulation section, the dimension of all the maps was maintained the same at 20 m × 20 m. The main parameters of these algorithms were the number of robots, which was three, a maximum number of iterations of 100 and a 1.5 m sensor range. Additionally, the number of obstacles and their positions differed from one map to another, but the same map was set for all algorithms. For example, map 1 and map 2 are different, but the same map 1 was employed to test all the algorithms and collect their data, with same for map 2, and so on. Each of the algorithms ran 30 times on each complex map, and the results are presented in Table 1 and Table 4.

It should be noted that in complex maps, CME could not complete 100 iterations and failed before getting to 60. Additionally, since it is deterministic, it will repeat the same pattern over and over unless the map changes to a lesser complexity. Moreover, because the other methods were able to complete 100 iterations, it would not be fair to calculate the *p*-value of the deterministic CME when it comes to exploration and time in less than 100 iterations, even though its exploration results were much less efficient than the other methods (Figure 7, Figure 8, Figure 9, Figure 10 and Figure 11).

Towards the end of the simulation, the obtained results showed how the proposed CME-SSA outperformed the other deterministic and hybrid methods on five different test maps. The average indicator demonstrates that CME-SSA performed better than the other methods on average exploration. On the other hand, standard deviations illustrate the stability of CME-SSA among the other methods. Moreover, the generated exploration *p*-values from the Wilcoxon rank-sum test demonstrate that CME-SSA ‘s superiority is statistically significant. As a result, these findings demonstrated the efficiency of the exploration of CME-SSA, and that is because the proposed method benefits from SSA exploitation, exploration, and convergence speed.

Because most of the *p*-values are less than 0.05, the Wilcoxon rank-sum statistical test findings demonstrate that the obtained results are statistically significant. The average and standard deviation show superior outcomes, demonstrating how effectively and reliably CME-SSA solves these challenges, especially in high-complexity maps.

Additionally, the run times reported in Table 6 show that the CME-SSA outperforms other algorithms on most test maps as it requires less time when exploring the same map compared with the other methods.

The lower mean values demonstrate that the CME-SSA outperforms others on average speed, and the lower standard deviations mean approximately every run needs the same amount of time to complete. The generated *p*-values of the time consumption by the Wilcoxon rank-sum test in Table 7 show the superiority is statistically significant. These findings demonstrated that the CME-SSA consumed less time to explore a map. That is another key factor accomplished and proved the high speed of the CME-SSA compared with the other similar methods.

The number of runs for the five complex maps in Figure 7, Figure 8, Figure 9, Figure 10 and Figure 11 is presented in Table 8. A single run that cannot complete 100 iterations successfully due to neighboring cells being occupied by obstacles or another robot is considered a failed simulation.

The number of uncompleted full runs of the proposed CME-SSA was zero for complex map 1, map 2, and map 3. For map 4 and map 5, it failed once and twice, respectively. Compared to other similar approaches, CME-SSA had the lowest number of failures when completing a full run. Finally, the third key was successfully achieved, besides space exploration and time consumption. Additionally, that shows how the proposed deterministic meta-heuristic CME-SSA delivered its best performance in five complex environments (map 1 to map 5).

### 4.4. Analysis Results Summary

The entire Section 4 results are summarized and collected as follows for the audience’s better understanding.

- Table 1 and Table 2 are the percentage of the explored area, and the *p*-value of their results is in Table 5.
- Table 2 and Table 6 are the time consumption results, and the *p*-value of their results is in Table 7.
- Table 3 and Table 8 are the numbers of failed simulations.

All comparisons between the proposed hybrid method CME-SSA and the referenced approaches: CME-GWO, CME-GWOSSA, CME-SCA, and CME, are presented in each of the above tables. In addition, the tables refer to the figures in which the simulations are observed. Additionally, the figures show the seven different maps and their complexity. The CME-SSA delivered a high space exploration, and the qualitative results showed that the lower mean indicator values demonstrate that CME-SSA outperforms others in exploration and speed averages, and the lower standard deviations demonstrate stability in exploration and time consumption. The generated *p*-values of the time consumption and exploration from the Wilcoxon rank-sum test show the superiority is statistically significant. Furthermore, the proposed CME-SSA method requires fewer trials to successfully complete a full run, whereas the other four methods need multiple attempts.

Another metric that determines the effectiveness of algorithms is their time consumption. The primary goal of any algorithm is to complete the desired task completed in the shortest amount of time. As a result, an algorithm that takes less time is considered energy efficient. The CME-SSA’s time consumption and the other three methods are computed for evaluation, and the results are recorded in Table 2 and Table 6. The results show that the proposed CME-SSA is computationally efficient because it takes less time to maximally explore the entire space. All the other methods take a much longer time and explore less space. The deterministic method CME in some maps was not even able to complete a full run, and since it is deterministic, the only way to solve this issue is to change the map, which is not possible in some cases.

### 4.5. Implementation on Hardware

It should be mentioned that the proposed method is implemented virtually, and simulation is conducted through MATLAB using the Robotic System Toolbox and Navigation Toolbox. To implement this method on hardware in the real-world, a Turtlebot [50] could be employed as a mobile robot with a Hokuyo laser range scanner [51] and a laptop with an installed Robotic System Toolbox to create the integration between MATLAB and the robot operating system (ROS) [52]. The reading laser sensor data with 240 to 360 degrees will go through MATLAB. The proposed method will calculate the next move and pass through the input sensor data. The system will have unknown measurement noise without using any external filter. A potential challenge could be the connectivity between the robot and the PC. However, that can be achieved via wireless routers with a strong Wi-Fi signal. The number of routers will depend on the indoor space size that needs to be explored. Several new frameworks designed a finite time that may be utilized to make the robot’s observation error uniformly constrained, as well as to reach a finite time convergence and reconstruct its external disturbances and uncertainties [53,54,55].

## 5. Conclusions

This paper proposed a new hybrid deterministic meta-heuristic method called CME-SSA for solving and optimizing the exploration problem in an unknown space environment using a multi-robot system. This system is based on a deterministic coordinated multi-robot exploration and the meta-heuristic salp swarm algorithm that mimics the swarming behavior of salps when navigating and foraging in oceans. The proposed method performed efficiently and improved the search space. Initially, CME takes care of the cost and utility values on the grid map. Then, the SSA efficiently selected the next move of each robot and improved the overall solution. A simulation was conducted over seven maps in a simple and complex environment and compared with four different methods: CME-GWO, CME-GWOSSA, CME-SCA, and CME. The results demonstrated the high performance of the proposed method in a different type of environment, with respect to three main key factors: high exploration rate, less time consumption, and also high success in navigating safely and avoiding obstacles in the workspace.

Future work will be conducted on multi-robot exploration based on a multi-objective metaheuristic algorithm to achieve two goals: the search for a new place and improving the map accuracy by avoiding the robot visiting an explored cell more than once.

## Figures and Tables

**Table 1:** Average of the percentage of the explored area after 30 runs, with 100 iterations each.

MAP	CME-SSA		CME-GWO		CME-GWOSSA		CME-SCA		CME	
	ave	std	ave	std	ave	std	ave	std	ave	std
Map 1	89.72	3.13	88.45	2.54	87.84	5.27	82.95	7.42	93.08	0.00
Map 2	96.22	2.58	91.75	3.28	87.63	7.47	88.49	8.32	97.31	0.00

**Table 2:** Average time taken to explore a map.

MAP	CME-SSA		CME-GWO		CME-GWOSSA		CME-SCA		CME	
	ave	std	ave	std	ave	std	ave	std	ave	std
Map 1	10.97	0.10	12.98	0.14	13.08	0.38	14.22	0.50	15.26	0.00
Map 2	11.01	0.29	13.12	0.24	13.10	0.25	14.59	0.78	13.40	0.00

**Table 3:** The number of failed simulations before completing 100 iterations of exploration on two simple environments (map 1, map 2).

MAP	CME-SSA	CME-GWOSSA	CME-GWO	CME-SCA	CME
Map 1	0	5	2	5	0
Map 2	0	3	2	8	0

**Table 4:** Avg and std of the percentage of the explored area in a complex environment for each algorithm.

MAP	CME-SSA		CME-GWO		CME-GWOSSA		CME-SCA		CME	
	ave	std	ave	std	ave	std	ave	std	ave	std
Map 1	92.84	2.62	85.57	11.57	87.31	9.11	87.13	8.95	39.24	0
Map 2	95.66	2.39	90.77	6.52	92.79	8.70	87.88	10.57	52.17	0
Map 3	95.20	2.52	88.80	8.11	87.45	7.68	87.04	8.70	36.48	0
Map 4	92.66	3.41	82.82	8.73	89.71	6.19	86.46	11.66	69.46	0
Map 5	96.34	1.90	73.42	12.23	70.34	14.48	65.30	15.93	54.23	0

**Table 5:** p-values for the exploration results in Table 1 and Table 4 as calculated by the Wilcoxon rank-sum test (N/A stands for not applicable, Null no result).

Map Type	Map No	CME-SSA	CME-GWOSSA	CME-GWO	CME-SCA	CME
Simple	Map 1	2.92 × 10^−9^	7.47 × 10^−10^	1.43 × 10^−8^	1.33 × 10^−8^	N/A
	Map 2	0.6411	1.21 × 10^−12^	1.21 × 10^−12^	1.21 × 10^−12^	N/A
Complex	Map 1	N/A	0.0138	0.0315	0.0035	Null
	Map 2	N/A	0.00	0.24	0.01	Null
	Map 3	N/A	0.00	0.00	0.00	Null
	Map 4	N/A	0.0261	0.0271	0.0451	Null
	Map 5	N/A	5.57 × 10^−10^	3.02 × 10^−11^	1.46 × 10^−10^	Null

**Table 6:** Time consumptions results of algorithms in the complex environment.

MAP	CME-SSA		CME-GWO		CME-GWOSSA		CME-SCA		CME	
	ave	std	ave	std	ave	std	ave	std	ave	std
Map 1	10.89	0.39	12.62	0.42	12.55	0.34	14.07	1.26	∞	0.00
Map 2	10.54	0.43	13.11	0.44	13.10	0.43	13.16	0.54	∞	0.00
Map 3	10.10	0.21	13.17	0.35	13.11	0.35	13.77	0.76	∞	0.00
Map 4	9.95	0.18	12.98	0.26	12.98	0.17	13.84	0.84	∞	0.00
Map 5	10.42	0.24	12.83	0.36	12.98	0.42	14.74	1.23	∞	0.00

**Table 7:** p-values for the time consumption results in Table 2 and Table 6 as calculated via the Wilcoxon rank-sum test (N/A stands for not applicable, Null, no result).

Map Type	Map No	CME-SSA	CME-GWO	CME-GWOSSA	CME-SCA	CME
Simple	Map 1	N/A	4.20 × 10^−10^	2.61 × 10^−10^	3.02 × 10^−11^	1.17 × 10^−7^
	Map 2	N/A	0.00	0.00	0.00	0.00
Complex	Map 1	N/A	0.00	0.00	0.00	NULL
	Map 2	N/A	0.00	0.00	0.00	NULL
	Map 3	N/A	0.00	0.00	0.00	NULL
	Map 4	N/A	1.21 × 10^−10^	2.37 × 10^−10^	1.96 × 10^−10^	NULL
	Map 5	N/A	1.10 × 10^−8^	6.72 × 10^−10^	3.34 × 10^−11^	NULL

**Table 8:** The number of failed simulations to complete 100 iterations of the hybrid exploration methods on five complex environment maps.

Complex MAP	CME-SSA	CME-GWOSSA	CME-GWO	CME-SCA	CME
Map 1	0.00	54.00	47.00	44.00	∞
Map 2	0.00	5.00	3.00	5.00	∞
Map 3	0.00	14.00	12.00	17.00	∞
Map 4	1.00	125.00	98.00	167.00	∞
Map 5	2.00	97.00	183.00	415.00	∞

## Data Availability

The data and code used in the research may be obtained from the corresponding author upon request.
